# Cardiac effects of direct anti-viral treatment in type II diabetic patients with hepatitis C infection

**DOI:** 10.1186/s12872-024-03973-1

**Published:** 2024-07-08

**Authors:** Khaled M. Elmaghraby, Lobna Abdel-Wahid, Yehia T. Kishk, Rania R. Y. Michael, Ahmed Abdel-Galeel

**Affiliations:** 1 Cardiovascular Medicine Department, Assiut University Heart Hospital, Assiut University, Assiut, Egypt; 2 Internal Medicine Department, Assiut University Hospital, Assiut University, Assiut, Egypt

**Keywords:** Diabetes, Sofosbuvir, Daclatasvir, Global longitudinal strain

## Abstract

**Background:**

The link between diabetes mellitus and chronic hepatitis C infection remains well established. It is estimated that up to one third of chronic hepatitis C patients have type II diabetes mellitus. Hepatitis C virus infection is one of the main global health burdens. Sofosbuvir and Daclatasvir are used as effective antiviral inhibitors of hepatitis C virus. The cardiovascular effects of those drugs are not well studied. We used electrocardiography and echocardiography with global longitudinal strain assessment by speckle tracking to detect their effect on cardiac function.

**Methods and results:**

One hundred diabetic patients with hepatitis C infection were included in the study. Abdominal ultrasound and laboratory work up were carried out for all participants. Left ventricular systolic and diastolic function were assessed by 2D-echocardiography and global longitudinal strain, before and 3 months after treatment. Results showed significant decrease in global longitudinal strain 3 months after therapy (-21 ± 4 vs. -18 ± 7; *P* < 0.001) but other echocardiographic findings showed no significant changes.

**Conclusions:**

Sofosbuvir and Daclatasvir were associated with early left ventricular systolic dysfunction as assessed by global longitudinal strain in diabetic patients. More deterioration in left ventricular systolic function was detected among those with Child-Pough class B. Further long-term follow-up may be required.

## Introduction

The link between diabetes mellitus and chronic hepatitis C (HCV) infection remains well established. It is estimated that up to one third of chronic HCV patients have type II diabetes mellitus [1]. Moreover, patients with diabetes are at increased risk of developing cardiovascular disease (CVD) with its manifestations of coronary artery disease, heart failure, atrial fibrillation, and stroke, as well as aortic and peripheral artery diseases [2].

HCV infection is one of the main global health burdens. It is one of the major leading causes of death and morbidity globally [3]. Recent estimates showed an increase in its seroprevalence over the last decade to 2.8% of the total population, corresponding to > 185 million infections worldwide [3]. The highest prevalence in the Eastern Mediterranean Region is 1.6% (1.4–1.8%). 290,000 (230,000–580,000) people die from HCV-related causes every year, and only 21% of people are diagnosed with HCV infection, and 62% of them receive treatment [4]. The Egyptian Demographic Health Survey (EDHS) in 2008 estimated HCV prevalence among the 15–59 years age group to be 14.7%. Accordingly, Egypt has the highest HCV prevalence in the world [5].

Regimens comprised of direct-acting antivirals (DAAs) that target different steps in the HCV life cycle have received breakthrough therapy status by the U.S. Food and Drug Administration (FDA), reducing the morbidity and mortality related to the disease progression [6] with improvement in liver function within 6 months of treatment compared to untreated patients [7].

On 24 March 2015, FDA and the European Medical Agency (EMA) added information to the Harvoni (ledipasvir / sofosbuvir) and Sovaldi (sofosbuvir) labels about the serious slowing of the heart rate of some anti-arrhythmic drugs when taken in combination with another DAA for the treatment of HCV infection [8]. After such FDA warning about the bradycardia occurring during DAA treatment, several concerns have arisen regarding cardiac toxicity, and a few cases of extreme bradycardia have been described. Furthermore, DAA therapy is often used in older populations which have a higher risk of heart related diseases, and consequently of related adverse events [9]. So, the possible exact cardiac effects of DAA therapy for HCV are not well studied. Moreover, no single study focused on cardiac changes following DAA assessed by tissue Doppler echocardiography.

### Aim of the work

We tried to study the actual effect of two commonly used anti-HCV treatment on cardiac structure and function in diabetic patients as assessed by electrocardiographic (ECG) and echocardiographic changes using tissue Doppler and speckle tracking imaging.

### Patients and methods

#### Study population

It is a prospective observational single center study that included 165 type II diabetic HCV infected patients who received a regimen of Sofosbuvir 400 mg and Daclatasvir 60 mg daily for 3 months in Assiut University hospitals [10].

Patients with prior history of ischemic heart disease, hypertension, bronchial asthma or chronic kidney disease (glomerular filtration rate below 60 ml/min/1.73 m^2^) were excluded. Also, we excluded patients with any significant abnormality in the baseline ECG, those with abnormal echocardiographic findings and those with history of malignancy or receiving cardiotoxic drugs. All patients were indicated to receive anti-HCV drug regimen (Sofosbuvir and Daclatasvir).

## Methods

All the study patients were subjected to the followings:

(A) At the time of recruitment:
  * Full history taking (focusing on demographic data and risk factors such as smoking and searching for possible exclusion criteria) and thorough clinical examination.
  * Abdominal ultrasound examination was carried out for all participants.
  * 12 lead ECG to report heart rate, PR and QT intervals and QRS-complex width.
  * Detailed echocardiographic examination including 2D-transthoracic echocardiography that was performed using Philips epic 7 echocardiography machine via single operator. All the measurements were performed according to American Society of Echocardiography/European Association for Cardiovascular Imaging (ASE/EACVI) 2016 guidelines and standards [11].

Evaluation of systolic function:

- = Assessment of left ventricular (LV) systolic function by determining ejection fraction (EF) by biplane Simpson’s method.
- = Global longitudinal strain (GLS) by speckle tracking:
  - *Image acquisition*: ECG gated 4-beat loops of three cardiac views (Apical 2-, 3- and 4-chamber views) were acquired while the patient is in the left lateral position and holding breath.
  - *Analysis*: It was done using latest Phillips ST package (Qlab10) and the acquired loops were selected according to the ASE/EAE guidelines [12, 13]. Automatic delineation of the endocardium and epicardium were carried on by the software. Manual adjustment of the borders was applied to include as much as possible of the region of interest and exclude the pericardium from the analysis for accurate assessment of the movement of the speckles. After that, automatic analysis was performed, and strain of each segment is spread in Bull's eye diagram from which the global strain can be calculated.

Evaluation of diastolic function:

- = Left atrial (LA) Volume: The measurement of LA volume was obtained using the apical 4-chamber and 2-chamber views using area-length method. Then left atrial volume index (LAVI) is calculated by dividing left atrial volume (LAV) in ml over body surface area (BSA) (m^2^) [11].
- = Mitral Inflow Patterns: Pulsed wave Doppler was performed in the apical 4-chamber view to obtain mitral inflow velocities for primary measurements of:
  - Passive ventricular filling in early diastole (E wave).
  - Late active filling phase during atrial contraction (A wave).
  - E/A ratio.
  - Deceleration time (DT).
  - Isovolumetric relaxation time (IVRT).

- = Tissue Doppler annular early and late diastolic velocities: Pulsed wave (PW) Doppler tissue imaging is performed in the apical views to acquire mitral annular velocities, primary measurements include:
  - The systolic annular velocity (S).
  - The early diastolic annular velocity (e´).
  - The late diastolic annular velocity (a´).
  - The mitral inflow E velocity to tissue Doppler e´ (E/e´) ratio.
    * Laboratory investigations: including liver function tests, mainly, serum albumin, bilirubin, and INR.

Child Pugh classification was determined for all participants [14].

(B) At follow up visit (after 3 months with completion of anti-HCV drug course):
  Three months later (immediately after completing antiviral regimen), all patients were subjected to ECG and detailed echocardiographic examination the same way as at the time of recruitment.

### Sample size calculation

Sample size calculation was carried out using 9 G*Power 3 software. A calculated minimum sample of 94 patients with chronic HCV infection was needed to detect an effect size of 0.3, with an error probability of 0.05 and 80% power on a two-tailed test.

### Ethical considerations

Written informed consent was obtained from all patients and the study was approved by the Ethical Committee of Assiut Medical School, Assiut university, under IRB number 17100173. The study was conducted in accordance with the Declaration of Helsinki.

### Statistical analysis

Data was collected and analyzed using SPSS (Statistical Package for the Social Science, version 20, IBM, and Armonk, New York). Continuous data was expressed in form of mean ± SD or median (range) while nominal data was expressed in form of frequency and percentage.

Chi^2^-test was used to compare the nominal data of different groups in the study while paired sample t-test was used to compare mean of continuous variables before and after treatment. Pearson correlation was used to determine the correlation between different continuous variables in the current study. Level of confidence was kept at 95% and hence *P* value was significant if < 0.05.

## Results

**Fig. 1:**
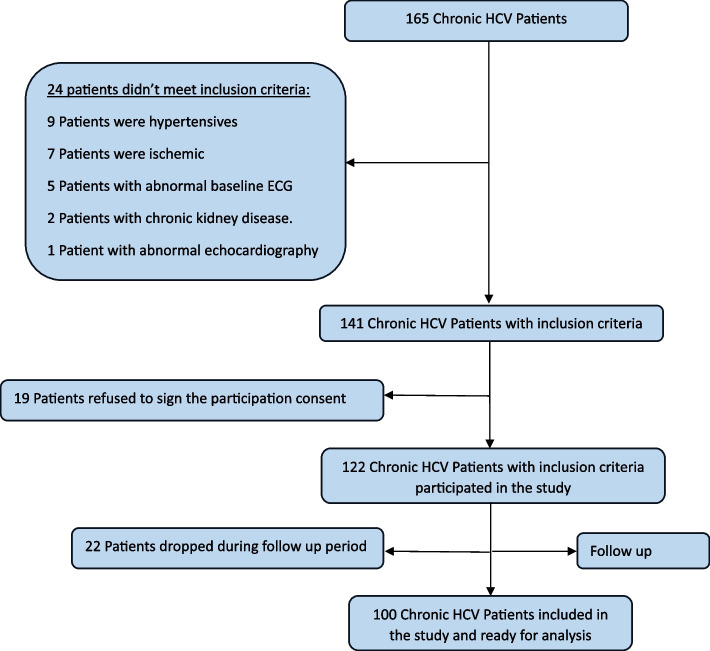
Flow chart of our study population

This study was performed at Assiut University Hospitals during the period between December 2018 to November 2019. We recruited 165 patients with HCV infection. Twenty-four patients didn’t meet our inclusion criteria. Nineteen patients refused to sign the consent for participation. Finally, 22 patients failed to complete the follow up period. Only 100 patients succeeded in completing the follow up visit, Fig. 1. The mean age was 52.59 ± 15.73 years old. 64% were males and 23% were smokers. The vast majority of our study population were in Child Pugh class A (87%), the rest were in class B, and no one was in class C, Table 1.

**Table 1:** Demographic, clinical and laboratory data of our study population

Variable	
Age (mean ± SD) in years	52.59 ± 15.73
Sex, male (%)	64 (64)
Smoking (%)	23 (23)
Duration of DM (mean ± SD) in years	7.73 ± 4.15
Child–Pugh class	
Child–Pugh class A (%)	87
Child–Pugh class B (%)	13
Child–Pugh class C (%)	0
HBA1c (mean ± SD) in %	6.39 ± 0.65
HB (mean ± SD) in mg/dL	12.8 ± 2.3
WBC (mean ± SD)	7875 ± 3540
Platelet count (mean ± SD)	185500 ± 20500
Serum albumin (mean ± SD) in gm/dL	4.25 ± 0.75
Serum bilirubin (mean ± SD) in mg/dL	0.95 ± 0.14
ALT (mean ± SD) in IU/dL	28.6 ± 1.5
AST (mean ± SD) in IU/dL	37.5 ± 2.4
Alkaline phosphatase (mean ± SD) in IU/dL	135.8 ± 45.9

*HB* hemoglobin, *HBA1c* glycated hemoglobin A1c, *WBC* white blood cells, *ALT* alanine transaminase, *AST* aspartate aminotransferase

The ECG and echocardiographic data of the study population before and after anti-viral treatment are displayed in Table 2.

**Table 2:** ECG and echocardiographic parameters before and after anti-viral course of treatment

Variable	Before treatment	After treatment	*P* value
Electrocardiographic parameters:			
Heart rate in beat/minute (mean ± SD)	79.84 ± 10.43	74.84 ± 8.58	0.08
PR interval in msec (mean ± SD)	157.57 ± 19.26	162.27 ± 15.81	0.07
QRS complex in msec (mean ± SD)	86 ± 5.47	89.05 ± 5.76	0.06
QT interval in msec (mean ± SD)	374.10 ± 21.03	387.50 ± 24.91	0.13
QTc interval in msec (mean ± SD)	401.35 ± 23.67	405.65 ± 26.48	0.09
Echocardiographic parameters:			
EF, in % (mean ± SD)	64.94 ± 5.10	63.03 ± 5.38	0.89
GLS in % (mean ± SD)	-21.7 ± 2.5	-18.5 ± 2.4	< 0.001
LAVI in ml/m^2^ (mean ± SD)	22.38 ± 7.24	23.96 ± 7.02	0.06
E/A ratio (mean ± SD)	1.22 ± 0.39	1.24 ± 0.38	0.47
DT in msec (mean ± SD)	181.60 ± 47.98	185.27 ± 39.27	0.37
IVRT in msec (mean ± SD)	92.35 ± 20.07	91.46 ± 17.92	0.63
e´ septal in cm/sec (mean ± SD)	12.16 ± 5.03	10.83 ± 3.24	0.37
e´ lateral in cm/sec (mean ± SD)	13.96 ± 4.28	13.76 ± 4.39	0.32
E/e´ ratio (mean ± SD)	7.28 ± 1.94	7.47 ± 2.34	0.08

*EF* ejection fraction, *GLS* global longitudinal strain, *LAVI* left atrial volume index, *DT* deceleration time, *IVRT* isovolumic relaxation time

**Fig. 2:**
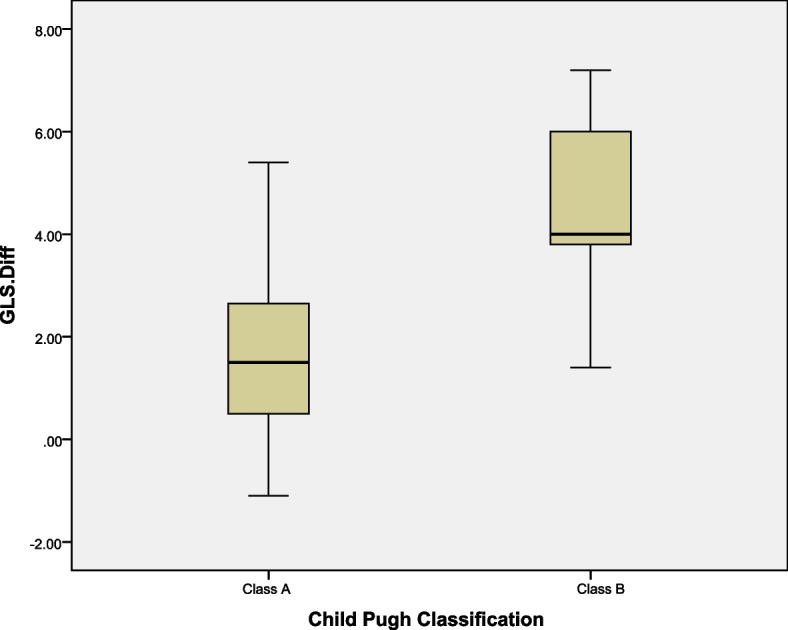
Distribution of GLS difference in our study population classified according to Child Pugh classification

Of note, there was no statistically significant change in T wave or ST segment after completion of anti-viral treatment. There was statistically significant difference regarding the change in GLS after treatment (-21.7 ± 2.5 vs -18.5 ± 2.4, 95% confidence interval for the difference -2.4—-1.7, *p* value < 0.001). This change was related to the baseline Child Pugh classification. Those with advanced liver disease (in our data those with Child Pugh class B) were more prone to have more deterioration in their LV systolic function as predicted by change in GLS after treatment, (1.61 ± 1.4 for class A versus 4.8 ± 1.7 for class B, *P* value < 0.001, 95% confidence interval -4.05—-2.30), Fig. 2 and Table 3.

**Table 3:** GLS distribution according to Child–Pugh classification before and after treatment

	Child–Pugh A	Child–Pugh B	*P* value
GLS before treatment (mean ± SD)	-21.46 ± 2.4	-23.66 ± 1.9	0.003^*^
GLS after treatment (mean ± SD)	-19.85 ± 2.29	-18.87 ± 2.47	0.156^*^
*P* value	0.001^**^	0.001^**^	
GLS difference (mean ± SD)	1.61 ± 1.44	4.79 ± 1.73	0.001^*^

*GLS* Global longitudinal strain

^*^Independent sample t test

^**^Paired sample t test

## Discussion

Viral hepatitis is estimated to be the 7th leading cause of mortality globally. The recent development of highly efficacious oral DAAs provides opportunities for reducing HCV disease burden and its onward transmission, with the potential for eliminating this blood-borne virus as a public health concern [15].

On March 24, the FDA issued a warning against taking the antiarrhythmic drug amiodarone in combination with HCV drug treatments and another DAA. In their announcement, the FDA stated that amiodarone “taken together with either the HCV drug ledipasvir/sofosbuvir or with sofosbuvir, taken in combination with another DAA for the treatment of HCV infection” can cause symptomatic bradycardia [8]. However, the long-term possible cardiac effects of DAA therapy for HCV are not well known especially among vulnerable group of our population such as diabetics. Given the firm relationship between HCV infection and coincidence of diabetes, our study tried to discover any possible cardiac effects of these commonly used anti-viral treatments. Moreover, to our knowledge, this the first study to address the possible effects of these drugs not only on cardiac electrical system but also on cardiac structure in diabetic patients. This was assessed using tissue Doppler examination of the LV.

Regarding ECG changes, our results didn’t find any statistically significant effect of these drugs on different electrical parameter. This is supported by Allam et al. [16] and Biomy et al. [17] who noticed no statistically significant difference at heart rate, PR interval, QRS duration, QT interval, QTc interval, ST segment and T wave after treatment when compared to their values before treatment. In a recent study conducted in Egypt using 24-h ECG monitoring during treatment, they reported that in non-cardiac patients receiving no cardioactive medications, the combination of sofosbuvir and daclatasvir for the treatment of HCV infection has no effect on heart rate, rhythm, conductivity, or heart rate variability. No symptomatic bradycardias, tachycardias, or syncope were reported or detected [18]. In a small sized study conducted in Italy, they concluded that in HCV patients treated with sofosbuvir and other DAAs, ECG parameter changes were mild and/or transient and did not translate into clinically significant electrophysiological effects in the absence of amiodarone coadministration [19].

In recent years, myocardial tissue deformation analysis by echocardiographic speckle-tracking imaging has provided new insights in the cardiac function assessment. LV-GLS, a measure of the LV myocardial systolic deformation over the longitudinal axis, has proved to be able to detect early LV systolic dysfunction in a variety of conditions, even when LVEF is still in the normal range [20, 21].

GLS can reflect early changes in LV systolic function at a stage when LV ejection fraction is still normal. The use of speckle-tracking GLS allowed the detection of subclinical LV systolic dysfunction. GLS-LV was independently associated with increased risk of future cardiovascular events [22].

In our study, we searched for early effects of DAAs on LV function. All patients in this study had received a combination of Sofosbuvir and Daclatasvir for 12 weeks.

Regarding LV systolic and diastolic function, in agreement to our results, Biomy et al. [17] and Mazzitelli et al. [23] observed no significant difference of LV systolic function before and after treatment when measured by biplane Simpson’s. In 2020. Ibrahim et al. reported that the current national protocol of HCV infection treatment with DAA agents used in Egyptian patients has a good cardiac safety profile. Such treatments have no effect on QTc interval, LV and right ventricular (RV) functions except for a decrease in RV-GLS in those with no liver cirrhosis and a reduction in lateral mitral E’ velocity in those with liver cirrhosis, both remained within the normal reference range [24]. In contrast to Ibrahim et al. results, our study reported a statistically significant worsening of left ventricular GLS in patients treated with DAA treatment for three months. This difference could be attributed to the nature of our study population who were diabetic, i.e., more vulnerable to cardiovascular adverse outcomes. In a report by Ahmad et al. six of thirty-four patients receiving IFN-free BMS-986094 regimes showed cardiotoxic changes; pathological analysis revealed severe myocyte damage with elongated myofibrils without gross necrosis [25].

Several studies have detected an association of HCV infection with cardiomyopathy, but no causal relationship or mechanistic link could be established so far [26–28]. The cardiovascular implications of HCV infections are incompletely explored, and possible mechanistic links are essentially lacking [29]. Multiple former studies regarding effects of HCV infection upon CVD risk produced ambiguous results. A recent meta-analysis of these studies concluded that HCV-infected patients have increased CVD-related mortality, carotid plaques, and cerebrocardiovascular events [30]. As a consequence of these studies, our study reported that the detected subclinical deterioration of LV systolic function as evidenced by GLS measurement was more obvious among those with impaired liver function as assessed by Child-Pough classification.

### Study limitations

The present study has some limitations. Our study sample size is relatively small, however, our claim is that we did study sample calculation before conducting the study which revealed that our study sample could be appropriate for drawing a statistically sound results especially with the use of appropriate statistical tests. The main study limitation was the short follow up period (only 3 months). However, we followed up our study population for three months immediately after completion of the treatment course. We suppose that better results could be achieved if we extend the follow-up period to 6 months. Clear effects of Sofosbuvir and Daclatasvir on cardiac function could be reported at 6 months for example.

## Conclusions

In diabetic patients with chronic hepatitis C infection, there is no actual effect of the two commonly used direct anti-viral treatment on the cardiac conductive system. Moreover, there is no obvious effect of the treatment on gross cardiac function. However, we observed a subclinical deterioration of LV systolic function as detected by GLS. More deterioration was detected among those with reduced Child-Pough classification. This worsening should raise the attention for using such drugs in diabetic patients with chronic hepatitis C infection and hepatic impairment.

## Data Availability

All data generated or analysed during this study are included in this published article.

## Abbreviations

ASE/EACVI: American Society of Echocardiography/European Association for Cardiovascular Imaging
BSA: Body Surface Area
CVD: Cardiovascular disease
DAAs: Direct-acting antivirals
DT: Deceleration time
ECG: Electrocardiography
EDHS: The Egyptian Demographic Health Survey
EF: Ejection fraction
EMA: European Medical Agency
FDA: Food and Drug Administration
GLS: Global longitudinal strain
HCV: Hepatitis C virus
IVRT: Isovolumetric relaxation time
LA: Left atrium
LAVI: Left atrial volume index
LV: Left ventricle
PW: Pulsed wave
